# Early, Patient-Centered, and Multidisciplinary Approach in Newly Diagnosed Multiple Myeloma: What Are We Talking About? A Case Description and Discussion

**DOI:** 10.1089/pmr.2021.0064

**Published:** 2022-01-04

**Authors:** Jonas Sørensen, Tanja Vibeke Sørensen, Kasper Hasseriis Andersen, Anne Dorthe Schou Nørøxe, Anne Kærsgaard Mylin

**Affiliations:** ^1^ Section of Palliative Medicine, Rigshospitalet, Copenhagen, Denmark.; ^2^ Department of Anesthesiology, Rigshospitalet, Copenhagen, Denmark.; ^3^ Department of Oncology, and Rigshospitalet, Copenhagen, Denmark.; ^4^ Department of Hematology, Rigshospitalet, Copenhagen, Denmark.

**Keywords:** caregiver issues, hematology-specific issues, hospital-specific palliative care issues, pain control, symptom assessment

## Abstract

Specialized palliative care (SPC) is a multidisciplinary need-based approach from the time a life-threatening disease is diagnosed. Patients with multiple myeloma (MM) will, at the time of diagnosis, often present with symptoms and needs that require a multidisciplinary approach. This case describes the course of a patient with newly diagnosed MM, involving all vertebrae and with no common analgesic treatment providing sufficient relief. High symptom burden and psychosocial and existential factors contribute to his total suffering. SPC, anesthesiological, and radio-oncological disciplines are integrated early, and the multidisciplinary approach includes support from social worker, psychologist, and physiotherapist. The needs and distress of the patients' wife are addressed. Barriers for further integration and the role of standardized care pathways are discussed, and the importance of systematic screening for symptoms and needs is highlighted. Integrating several disciplines may be a prerequisite for antineoplastic treatment being initiated for patients with newly diagnosed malignant disease.

## Introduction

Historically, specialized palliative care (SPC) has been considered relevant only for cancer patients in the terminal stage of the disease and after tumor-directed treatment has ended. However, the Danish Health Agency and international sources emphasize that palliative care must be offered from the time of diagnosis as a multidisciplinary relief and support effort and delivered need based throughout the trajectory.^1^

Palliative care is delivered at different levels. Primary palliative care is often described as “community-based” and is delivered from family physicians or in nursing homes or delivered in a general medical/surgical hospital ward. Palliative care delivered by an oncologist or hematologist is often referred to as “secondary palliative care” and includes palliative and supportive care efforts delivered concurrently with the active treatment the patient receives. This may include analgesic treatment, management of, for example, skin toxicities, nausea or insomnia, advice from a dietitian or physiotherapist, an initial advance care planning conversation, or referral back to primary palliative care in the community.

In the event of a large symptom burden and complex psychosocial issues or existential distress, patient and family support can be provided through a multidisciplinary SPC team (tertiary level),^2^ often with an in-patient service and, therefore, hospital based.

Patients with multiple myeloma (MM) are often hospitalized at the time of diagnosis or receive initial treatment during hospitalization. They are known to have a high symptom burden, reduced quality of life (QoL), and affected functional status. A large proportion of patients with MM will need analgesic treatment, and they often present with needs that require a multidisciplinary approach.^3,4^ Documented effects of early integration of SPC for hospitalized patients with MM are sparse. However, the effect of integration of SPC for other hospitalized patients with hematological disease is documented. Integration of SPC for patients undergoing hematopoietic stem cell transplantation (HSCT) has shown positive effects in a recent randomized clinical trial. Among enrolled patients hospitalized for HSCT, the management of physical and psychological symptoms showed significantly less decrease in QoL, less increase in depression, lower anxiety, and generally less increase in symptom burden during the first two weeks, compared with a control group. Even at three months after HSCT, intervention patients versus control group patients had significant higher QoL scores and fewer depression symptoms.^5^

## Case Description

A 49-year-old man, previously healthy and working as a police officer, is diagnosed for back pain for three to four months. Within a few weeks, he is reporting fatigue, nausea, confusion, constipation, and weight loss of 10 kg. A magnetic resonance imaging (MRI) column shows pathological signal changes in all vertebrae and the pelvis, and he is admitted to the department of hematology. Detection of an M-component and clonal plasma cells in a bone marrow biopsy results in the diagnosis MM. Myeloma-defining events include advanced bone disease with widespread bone destruction, hypercalcemia, and renal insufficiency. Because of a severe pain condition with both nociceptive and neuropathic components, transdermal fentanyl, subcutaneous pro re nata (PRN) oxycodone, and gabapentin (adjusted to renal function) are added.

Specialized palliative interconsultation is requested on day 4 of admission. The patient is screened, as part of the standard routine, with the EORTC (European Organization for Research and Treatment of Cancer) QLQ C15-Pall symptom screening tool, which is an abbreviated 15-item version of the EORTC QLQ C30—a cancer health-related QoL questionnaire that is widely used in clinical trials and investigations using patient-reported outcome measures (PROMs) for individual patient management.^6^ Besides severe pain, the patient reports reduced level of function, insomnia, weakness, constipation, fatigue, tension, and a QoL score of 4 (1 = very poor, 7 = very good). For the neuropathic pain, amitriptyline is added. After radio-oncological advice, the patient is started on palliative radiation therapy (RT) on selected vertebrae.

Despite increased dose of slow-release opioid and adjuvant analgesic treatment over the next few days, the patient requires considerable amounts of PRN oxycodone administered subcutaneously. The patient has not had any continuous sleep for several days, and he now confirms visual hallucinations. Nonpharmacological and pharmacological treatments of delirium are initiated. Some effect from the RT is expected within days, but despite subcutaneous syringe driver with oxycodone 200 mg + PNR oxycodone (on what is, in total, equivalent to 1000 mg p.o. morphine per day), the patient is not sufficiently relieved. A possibly hyperexcited state with opioid-induced hyperalgesia is suspected, and because of the known N-methyl-D-aspartate (NMDA) antagonist properties of methadone, the patient is rotated to methadone.^7^

In the setting of the hematological ward, the patient is titrated to 30 mg methadone i.v. over the course of about three hours. After this, the patient is clearly relieved, and on the numeric rating scale pain score, he reports a decrease from 10 to 4, without significant sedation. He is now so calm he can move in bed and speak over the phone with his wife. The rotation to methadone is handled by a specially trained palliative nurse from the SPC team, with guidance from a palliative physician. This setup outside an SPC ward or anesthesiology ward demands great teamwork with the hematological staff to ensure close observation of the patient in the subsequent hours and days.

A new MRI column confirms suspicion of further collapse of vertebrae, including Th5, and a total reduction of 13 cm (5 inches) is evident. New vertebrae are, therefore, included in the RT beam field. Over the next day, the patient becomes increasingly bothered by pronounced myoclonus due to high-dose opioids. All of this contributes to poor sleep and a high degree of anxiety, complicating his existential suffering. A daily dose of methadone 60 mg on an intravenous syringe driver is required for pain relief. However, on delivery of these doses, short periods of apnea begin to occur, and after 24 hours, the intravenous syringe driver with methadone is paused.

Balancing pain relief and sedation is not possible, and the difficult situation is discussed in a multidisciplinary group consisting of the senior hematologist, the anesthesiologist, and the palliative physician on call. The patient is never critically sedated or unstable, but that evening he is transferred to the intensive care unit with the intension of adding s-ketamine for better pain relief and to achieve sleep. However, the added s-ketamine has no good analgesic effect. Then, despite very difficult pathoanatomical conditions, an epidural catheter is placed with bupivacaine/morphine, with good effect. The patient is once again relived and without apnea.

The next day the patient is transferred back to the hematological ward. Over the next two weeks, the planned course of RT is completed, and systemic antimyeloma treatment is continued. Crucially aided by the epidural local analgesia, slow-release systemic opioid and adjuvant analgesic treatment is gradually titrated, and the patient takes part in the in-patient rehabilitation program. Concurrently, the patient and family are in contact with the SPC team for full multidisciplinary support. The wife is screened for needs and found to have a high degree of distress and specific needs in relation to the family's situation and her work life.

Both the patient and his wife are on sick leave, and at home they have three children aged 11–12 years. Through the SPC team, the couple receive counseling from a social worker and a psychologist. Besides consultation with a palliative nurse and a physician, the patient receives advice from a physiotherapist affiliated with the SPC team, bridging the rehabilitation program to the community setting when he is discharged. On a routine EORTC QLQ C15-Pall symptom assessment 10 weeks after discharge, the patient reports pain as being “quite a bit,” and a QoL score of 4. All other symptoms are reported as “mild” or “none at all.”

In the department of hematology, the patient has later undergone high-dose chemotherapy with stem cell support, and he is still seen on an out-patient basis by the SPC team. Analgesic treatment consists of p.o. methadone, pregabalin, and low-dose amitriptyline. Screening at regular intervals for symptoms and needs guides the extent and nature of the SPC support.

At present, the patient lives a reasonably active life with his family, and he is back at work part time.

## Discussion

This case description emphasizes the importance of implementing a coordinated multidisciplinary approach from the time of diagnosis, to assist a severely afflicted patient and a profoundly affected family. It is an example of how a collaborative effort, at the right time, is a prerequisite for beneficial patient outcome in newly diagnosed hematological patients with complex needs. Taking a multidisciplinary approach in cancer care is increasingly seen as part of standard care provision, backed by a growing body of evidence, and strongly supported by clinical consensus.^8^ The World Health Organization (WHO), defines “integrated service in health care” as “the organization and management of health services, so that people get the care they need, when they need it, in ways that are user-friendly, achieve the desired results and promote value for money.”^9^

However, the timely integration of different disciplines is complex, and specifically for SPC, several barriers to integration have been suggested, including organizational structures, inadequate resources and funding, and a lack of education and training; but misconceptions about the term “palliative care” may also hinder integration.^10^ Thus, such barriers must be considered and incorporated in future research strategies regarding the integration of palliative care.^11^ Yet for those seeking to successfully deliver supportive and palliative care from the time of diagnosis, and doing this alongside tumor-directed treatment, there are validated instruments available to identify symptoms and unmet needs.

The use of PROMs is a cornerstone in delivering patient-centered care, and international organizations encourage their use.^12,13^ SPC has a long history of systematically using PROMs, enabling the SPC team to address patients' needs along the disease trajectory. Nevertheless, the infrequent and unsystematic use of PROMs in standard oncology or hematology care is described as a major factor underlying inadequate symptom relief,^14,15^ and that may unintentionally delay the integration of other disciplines, including SPC.

Standardized care pathways have been proposed as an integrated care model, in the attempt to solve the challenges of operating complex care processes where different professions and competencies must work together in a formalized structure.^16^ Disease-specific quality indicators and defined timelines in care can provide a coarse matrix for a patient's treatment trajectory. However, to achieve truly patient-centered care, the use of patient-reported data and the systematic screening for symptoms and needs are mandatory elements. Acknowledging that needs differ from one individual to another, and over time, both justify and challenge the road toward implementing standardized care pathways. The role and the nature of these pathways call for further investigation.^2^

Early SPC not only results in better symptom management, with significantly improved patient outcomes including pain relief and reduced anxiety.^17^ For patients as well as informal caregivers, early SPC has also shown significant reduction in total distress (HADS-total).^18^ Besides these obviously important beneficial patient- and caregiver-centered outcomes of early SPC, there might be another crucial point to make, as illustrated by this case. Newly diagnosed patients with complex symptomatology and high levels of physical suffering and distress may not be capable of receiving, or in a performance status that allows them to receive, the intended life-saving or palliative treatment.

Thus, the integration of several disciplines may be a crucial prerequisite for initiating disease-specific treatment. In this case, because of the early integration of hematological, SPC,^1,2,16,17,19^ anesthesiological,^20^ and radio-oncological^21^ disciplines, the health system was able to create the best possible conditions for relief, support, and rehabilitation.

## Abbreviations Used

EORTC: European Organization for Research and Treatment of Cancer
HSCT: hematopoietic stem cell transplantation
MDEs: myeloma-defining events
MM: multiple myeloma
MRI: magnetic resonance imaging
NRS: numeric rating scale
PRN: pro re nata
PROMs: patient-reported outcome measures
QoL: quality of life
RT: radiation therapy
SPC: specialized palliative care
WHO: World Health Organization
